# Clinical Lectures

**Published:** 1870-07

**Authors:** Jno. E. Owens

**Affiliations:** Surgeon to the Hospital (*Thrombosis and Aphasia*)


ARTICLE V. — *Clinical Lecture.* Delivered at St. Luke's Hospital. By JNO. E. OWENS, M. D., Surgeon to the Hospital (*Thrombosis and Aphasia.*)

John W. Wilson, æt. 32, born in England, a book-keeper, was admitted September 29th, with a simple diarrhœa, with which he was attacked two or three days before admission, and which yielded in 24 hours after the administration of pills, consisting of Acetate of Lead, grs.ii, Powd. Opium, gr.j. I saw him on Sunday morning, when he commenced taking the pills. On Monday morning he took the fourth pill, and the diarrhœa being checked, the medicine was discontinued. With the exception of a very slight debility in consequence of his indisposition, he felt very well and comfortable, and would have been discharged on Tuesday, had he not been attacked, on Monday evening, with a pain in the lower part of the right leg and the corresponding foot. This, with marked symptoms of lowered vitality, rapidly increased, until mortification set in. The heart and lungs sounds were natural.

Friday, Oct. 5th. The greater part of the foot was of a dark purple color, indeed, in some places it was almost black. At this time the late Prof. Brainard was good enough to see the case with me.

Sunday, Oct. 7th. I received a note from the hospital, saying that the patient had not spoken for an hour. At six o'clock in the morning, he called the nurse and asked him if he intended to get up soon. A few minutes afterwards, the nurse, upon going to the ward, found that the patient mumbled his words so much, he could not make himself understood. Upon calling about 10½ o'clock, A. M., I found that he had a partial paralysis of motion and of sensation of the right side. He could roll the arm very slightly. The left corner of the mouth was drawn towards the corresponding side; he was unable to move the tongue in any direction; the pupils were normal; the skin was in good condition; the pulse 90, heart and lung sounds natural, and appetite good.

Monday, Oct. 8th. There is tenderness over the large arteries, in the arms and legs; the paralysis is decreasing; the patient thrusts out the tongue, and, with some difficulty, moves it from side to side; the left corner of the mouth is no longer drawn to one side; the arm and leg are very feeble. Although he can now use several words, he is unable generally to communicate his ideas. He managed to say, "That'll do." The bowels have twice moved, in response to Pil Cathart Comp. Up to this time, the patient has been taking 30 drops of the following mixture:—Tr. Ferri Chlorid ℥j, Quiniæ Sulph. grs.xxx, three or four times, daily; anodynes in the shape of Ext. Hyoscyami; from 3 to 6 grs., or Morphiæ Sulph. gr.ss., repeated pro re nata; egg-nog, and the most nourishing food.

Tuesday, Oct. 9th. Urine normal; has very good motion of the right arm and leg; heart sounds normal; temperature of two sides of the body about the same.

Wednesday, Oct. 10th. Leg tense, mortification extending; pulse 96. For the last three days, the answer to almost every question has been, "Yes, yes, yes," whether it was a proper answer to the question or not. When restless, the following pill was given:—Morphiæ Sulph. gr.ss., Gum Camph. grs.jj.

Thursday, Oct. 11th. Had a chill this morning; pulse 119; appetite not quite so good; treatment continued.

Tuesday, Oct. 16th. Chill in the evening; ordered additional Whisky, Quiniæ Sulph. grs.jjj, every three hours, Sulphite of Lime, grs.x, in milk, every hour or two, with Morphiæ Sulph. at night; appetite poor.

Thursday, Oct. 18th. Mortification extending; pulse 96. The patient knows his age, (32), but when asked he can only say, "Thir," and shows vexation from his inability to answer perfectly. He was, you'll remember, born in England, and when I told him that he was born in France, he said, "No, no." The Quinine pills were discontinued, and the mixture of Iron and Quinine substituted.

Friday, Oct. 19th. Had a chill this morning; pulse 150; appetite failing; in other respects the same. Friday evening, pulse 96.

Saturday, Oct. 20th. The double-flap operation was performed above the knee. After the operation we gave the patient Morphiæ Sulph. gr.i., Gum Camphoræ, grs.ij.

Sunday, Oct. 21st. He rallied very well yesterday, from the operation; but did not sleep till about four o'clock this morning; pulse 130 and feeble, hands cold and clammy. Prescribed egg-nog, beef tea, soft-boiled eggs, and ℥ss. three or four times daily, of the following: Tr. Ferri Chlor. ʒiij., Quiniæ Sulph. ʒss., Sacch. Alb. ʒij., Aquæ ℥vj. et ʒv. The patient has slept a good deal since four o'clock. The nose looks pinched; face is much thinner, since yesterday.

Monday, Oct. 22nd. Patient continued to sink since yesterday, and died at 1½ P. M.

GENTLEMEN: At our last meeting, so much time was taken up with the operation, that we were obliged to defer, till to-day, our comments upon this very important and interesting case. Had the patient been a man of advanced age, or had he been a rheumatic or gouty subject, or excessively debilitated from long continued wasting disease, the condition under which he labored, and which has terminated so fatally, would have lost much of its rarity, and our knowledge of its pathology might have been more satisfactory. But when we consider that the history of this case does not point to any of the above conditions,—that he was splendidly developed and well nourished, we must look for other and more insidious causes, and congratulate humanity, that, at least, some of our most fatal diseases may make most extensive ravages, and entail upon us comparatively little suffering. Our patient seems to have had, throughout, only about three days of real suffering. Let us, once more, glance at the symptoms presented by this case, together with its history, and see how far a consideration of them may lead us to a correct diagnosis. The diarrhœa, I think, we may set aside, as having no bearing upon the case whatever.

On Monday evening, Oct. 1st, the patient was attacked with a pain in the leg and foot. This pain came suddenly and was tolerably severe, during the evening and night. On Tuesday when I saw him it had increased, so that anodynes were necessary to allay it. The foot and lower part of the leg presented a dusky appearance, the temperature was much lowered, and the blood coursed very sluggishly and lazily through the veins on the dorsum of the foot. When a part is about to undergo the process of mortification, there is generally an aggravation of all the previous symptoms. The pain and sensibility become more intolerable, the dusky hue changes to a mottled appearance, the tension becomes greater, and here and there blisters arise, which contain serum more or less tinged with the coloring matter of the blood. Now, gentlemen, when a limb presents this appearance, its nature is unmistakable, and unless the cause can be promptly removed, the death of the entire part is inevitable. It may be well, here, to mention a case illustrative of the fact that a part, presenting the appearance just described, may return to its normal condition, if the cause be promptly removed. For the facts of the case, I am indebted to Prof. Andrews. From some cause, which I do not now remember, an arm became enormously swollen. The tension was so great, that pressure upon the arteries of the limb, so interfered with the nutrition of the part, by cutting off the circulation, that mortification was about to commence, or indeed, if I remember correctly, it had in some parts of the limb already taken place. The part was of a dark purple, cold and blistered. Free incisions into the limb promptly removed the cause, the circulation became re-established, and the process of mortification stopped. So by the timely action of the experienced surgeon, a limb was saved—indeed, perhaps the patient's life. Physicians may boast of their alteratives, but it has been said that none of them are equal to the scalpel.

The complete cessation of vitality is denoted by a purple or black appearance of the part, the absence of all heat and sensibility, and a peculiar fetid odor. In general, there is more or less crepitation upon pressure, but in this case I could not detect any. The constitutional disturbance, which is generally of a typhoid character, was in this case very slight. The pulse, which usually in such cases runs up to 140 or 160, except on two or three occasions, and then only for a short time after the chills, did not go above 96.

But, gentlemen, were you called to a case of this kind early, what would be the nature of your examination of the part? In the first place, you must inquire whether the pain, which came on so suddenly in the leg, exists in any other part of the body. You find that it does not. It is not likely, then, that you have rheumatism to deal with. Taking in the general appearance of the leg, you will, either at your first or your second visit, notice the dusky color of the foot and more or less of the leg. You press upon the surface and find that the blood, which has been displaced by the pressure, returns but very slowly and lazily to the spot, and in the same manner does the blood course through the veins upon the dorsum of the foot. Having now taken note of the pain, and the character of the local circulation, your attention must be directed to the temperature. The toes, and perhaps the whole foot, are quite cold. The part will likely pit on pressure. You will at once determine from this condition of sluggish circulation, lowered temperature, and pitting on pressure, that the vitality of the part is very much lowered. Now when the vitality of a part is excessively depreciated, the part may die. Your attention would then be directed to the arteries through which the limb receives nourishment. These vessels you may examine very easily, if there is not much swelling. The arteries to be examined in the foot are the dorsalis pedis, midway between the two malleoli, and the posterior tibial, where it passes, immediately behind the internal malleolus.

Gentlemen, if you find the above condition existing, and no pulsation in these arteries, there is not the shadow of a doubt as to the nature of the case. You have diagnosed, then, approaching mortification, and if you are a young man, just at this point, although you may thoroughly understand the nature of the case, you will have a consultation. The prognosis in such cases is unfavorable.

Being a young man, its result might be laid at your door. Hence, it is best to divide the responsibility of these cases, otherwise your future prospects might be sadly impaired. The mortification, of which we have been speaking, is, however, only a symptom of some deep-seated and insidious disease. Our object will next be, to find out the nature of this disease—the cause of the gangrenous leg. The causes of mortification of an extremity are, defective nervous energy, constitutional debility, mechanical obstruction, ergot of rye, traumatic injuries, and diseased blood vessels. Now let us endeavor to select from this list a cause for our patient's trouble. In paraplegia, large sloughs sometimes occur on the nates and about the gluteal region and hip. These are often mistaken for bed-sores; but I do know that these sloughs occur in regions where there is not sufficient pressure to cause them. Such a condition has been lately exemplified in a patient now in the hospital. In such cases the application of a blister is frequently followed by extensive sloughing. According to Gross, the division of the peroneal nerve, in the removal of a tumor, has been followed by mortification of the small toes. These are examples of mortification from defective nervous energy. It is true that the patient had a hemiplegia, an example of defective nervous energy, but this could not have caused the gangrene, because the paralysis did not occur until nearly a week after the mortification had set in. There was no injury to the nerves of the part. Defective nervous energy did not cause it. Neither was it caused by constitutional debility, for you may all remember the splendidly developed shoulders and chest, and the well nourished face. By mechanical obstruction, we mean tight bandaging, inordinate swelling, pressure by a tumor on the blood vessels, ligature of an artery, etc., none of which existed. In some parts of Europe, mortification of the fingers, indeed all the remote parts of the body in some cases, has prevailed, endemically, from the use of rye bread, in the flour of which was mixed the secale cornutum, or ergot of rye. It is very rare in this country. I am not sure that there is a single case. There is nothing in the history of our case to point to this cause. This species of mortification is dry, black, and free from odor; whereas that of our patient was moist, and very offensive. Of course, in this case, we must also set aside traumatic injury. In seeking a cause for this trouble, let us for a moment consider diseased blood vessels. The blood vessels, I need scarcely tell you, are the arteries, veins and capillaries. Phlebitis, or simple inflammation of the veins alone, has never in my experience led to mortification of an extremity, nor have I ever read of such a case. What it might do were time allowed, I am unable to say, but such cases usually run a rapid course and end unfavorably from pyæmia. We have now reasoned somewhat by exclusion, which has left us, as a cause of the mortification in our case—diseased arteries. In old age, mortification sometimes takes place from ossification of these vessels, but you must remember that our patient was only 32. Had he been afflicted with chronic gout or rheumatism, there might, possibly, have been a calcareous degeneration of the coats of the arteries, or of the heart, even at his age. If the patient has had one attack of rheumatism you will be led to an examination of the blood vessels and heart. These deposits usually occur about the valves of the heart and aorta, leading to more or less functional disturbance of this organ, and perhaps always to abnormal valvular sounds. The ossific deposit may often be felt in the radial arteries at the wrist. His heart sounds were, however, normal, and the arteries at the wrist felt soft and natural. Our idea of the pathology of the case was not, then, one of ossific deposit in the walls of the arteries.

Now, gentlemen, I have been telling you what was not the pathology of this case, and as far as my time would permit, the manner of tracing the real cause; let me now tell you what was the cause of it. Rudolf Virchow, the distinguished pathologist of Berlin, has described in his book upon Cellular Pathology, an affection, which he terms "Thrombosis"—an affection which seems not to be thoroughly understood, but, upon which much new light must very soon be thrown. Thrombus means a clot, and is derived from the Greek word, *thromboa*, which means to coagulate. The whole process I told you is comprehended under the term Thrombosis, by which you will understand that the artery, or vein, or indeed both of them, are plugged up with a clot of blood. This was our diagnosis before death, and the only satisfaction that now remains is the confirmation of our diagnosis by the pathological specimen—part of the popliteal artery and vein, both of which are occluded by coagula. The cause of Thrombosis is, as yet, obscure. The autopsy, in this case, does not seem to throw much light upon the subject, especially as we were not permitted to examine the body as thoroughly as desirable, because of the near approach of the hour of burial. The vein is inflamed, and the artery heightened in color. Whether the inflammation led to the coagula, or the reverse, I am unable to determine positively. I am inclined to the former view of the case. Since the patient's death, the woman with whom he boarded told us, that she frequently heard him complain of soreness and pain, when he pulled on his boots. This seems to favor the idea of a pre-existing phlebitis. Virchow, however, says: "That a phlebitis affects the walls, and not the contents of a vessel; that, in large vessels, the most different layers of their walls may enter upon every phase of inflammation, and yet all the time their channel remain entirely unaltered; that even abscesses may form, which cause the walls to bulge on both sides, without any coagulation of the blood ensuing in the cavity of the vessel." It does not seem to me that this latter condition could exist without great danger of Thrombosis. Such an eminent authority as Virchow, however, is entitled to much respect. Pathological research only will reveal the subject in its true light.

There is one other point to which I would call your attention; and that is, to the propriety of making incisions in the mortified limb in order to let the poisonous and irritating fluids drain off. This was suggested at the consultations, but it was decided to substitute a blister upon the calf of the leg, that more or less of the poisonous fluids might pass off in that way. This practice, though quite common in this class of cases in France, has not, it seems, obtained in this country. An examination of the limb has settled the question in my mind that it is proper to make incisions and let out the pent-up secretions. When I opened the leg, nearly a quart of dirty, sanious, highly offensive and irritating fluid came away, which, when left in the limb, I am satisfied, does harm. This quantity remained in spite, too, of considerable fluid having drained off through the blister. Do not hesitate, then, to make incisions instead of blistering. The leg was amputated above the knee, without waiting for a line of demarcation, because it was thought that amputation would give the patient the best chance for recovery.

Autopsy: Lungs hypostatically engorged; liver, heart and kidneys normal in appearance; circle of Willis very much attenuated; the brain was softened; but we will say more of the latter when we come to speak of the paralysis and of the aphasic condition of the patient.

(Continued in the next.)

